# The Long-Term Impact of Polysaccharide-Coated Iron Oxide Nanoparticles on Inflammatory-Stressed Mice

**DOI:** 10.3390/jox14040091

**Published:** 2024-11-07

**Authors:** Julia Göring, Claudia Schwarz, Eric Unger, Rainer Quaas, Ingrid Hilger

**Affiliations:** 1 Experimental Radiology, Institute of Diagnostic and Interventional Radiology, Jena University Hospital, Friedrich Schiller University Jena, Am Klinikum 1, D-07747 Jena, Germany; julia.goering@med.uni-jena.de (J.G.);; 2 Chemicell GmbH, Eresburgstrasse 22-23, D-12103 Berlin, Germany; info@chemicell.com

**Keywords:** iron oxide nanoparticles, polysaccharides, nanomedicine, long-term toxicity, NF-kappaB, long-term biocompatibility, theranostic, metabolism, health

## Abstract

Since iron oxide nanoparticles (IONPs) are expected to be important tools in medical care, patients with inflammatory diseases will be increasingly exposed to IONPs in the future. Here, we assessed the short- and long-term impact of polysaccharide (PS)-coated IONPs on mice with persistent systemic inflammation. To this end, PS-IONPs were synthetized by a core-shell method. Mice were regularly injected with sterile zymosan. PS-IONPs were administered intravenously. At specific nanoparticle injection post-observation times, the organ iron concentration was determined via atomic absorption spectrometry, the expression of NF-κB-related proteins using SDS-PAGE and immunoblotting, as well as body weight and haemograms. Finally, the mediator secretion in blood plasma was analysed using multiplexed ELISA. Our data show that PS-IONPs induce short-term changes of iron levels in distinct organs and of NF-κB p65 and p50, p100, COX-2s, and Bcl-2 protein expression in the liver of inflammatory stressed mice. In the long term, there was an attenuated expression of several NF-κB–related proteins and attenuated features of inflammatory-based anaemia in blood. PS-IONPs weakly influenced the blood cytokine levels. PS-IONPs are biocompatible, but given their short-term pro-inflammatory impact, they should prospectively be applied with caution in patients with inflammatory diseases of the liver.

## 1. Introduction

Iron oxide nanoparticles (IONPs) have been incorporated into medical care due to their unique properties, such as superparamagnetism, biocompatibility, and the ability to receive a coating. Moreover, the superparamagnetic properties of IONPs make them attractive for magnetic resonance imaging, drug delivery, and cancer therapy applications (e.g., [1,2,3,4,5]). For these reasons, their administration to healthy and morbid patients is almost a matter of fact in the clinical setting. In this view, their administration particularly to individuals with inflammatory diseases is of consideration, since these disorders are globally very prevalent [6]. Despite of it, very little is known on how nanoparticles influence health conditions of people with inflammatory diseases over time.

To improve their biocompatibility and enhance dispersibility, IONPs have been provided with specific surface coatings (e.g., [7,8]). In particular, the employment of cross-linked polysaccharides is beneficial in terms of enhanced nanoparticle accumulation in target cells, the modulation of specific surface properties, and the formation of protective monolayers (e.g., [9]).

Upon intravenous injection (i.e., the most common drug application route), IONPs (and nanoparticles in general) interact easily with macrophages, which play a crucial role in their clearance from the blood circulation. Upon cellular uptake by macrophages, PS-IONPs undergo intracellular degradation, and this event can potentially dysregulate cellular iron levels, induce oxidative stress, and enhance the expression of pro-inflammatory cytokines in target cells [10,11]. This particularly applies when the intracellular formation of reactive oxygen species overflows the capacity of iron binding proteins of the cell [12]. This can lead to oxidative stress, which may even influence bone marrow cell function and remodelling [13]. Furthermore, the intracellular delivery of polysaccharides during nanoparticle degradation is to be taken into account, since they play a role in the immune system’s regulation (e.g., [14,15,16]) on themselves. In particular, they may stimulate immune cells to produce pro-inflammatory cytokines such as TNF-alpha, IL-1, and IL-6, which may finally exacerbate inflammatory responses, particularly in chronic inflammatory conditions or infections [17]. Additionally, polysaccharides can interact with pattern recognition receptors (PRRs) on immune cells, triggering signalling pathways that promote inflammation, such as the activation of the nuclear factor kappa B (NF-κB) pathway [18]. They also contribute in causing overproduction of nitric oxides (NO) in macrophages. While NO has protective roles, excessive amounts can contribute to tissue damage and systemic inflammatory disorders, such as sepsis [15]. Continuous stimulation of immune cells by polysaccharides may lead to chronic inflammation. For instance, prolonged exposure to certain polysaccharides may keep macrophages in an activated state, resulting in a sustained release of inflammatory mediators [19]. Therefore, a specific balance is essential for maintaining immune homeostasis and preventing excessive inflammation.

While in vivo long-term effects of polysaccharide-IONPs (PS-IONPs) have not been reported so far, there are studies with mice describing the effects of nanoparticles in general (e.g., [20]). In particular, in vivo long-term effects of PS-IONPs can vary depending on the specific type of nanoparticles, dosage, exposure duration, and the physiological response of the individual hosting them. All this information is important for determining the benefits of PS-IONPs and the potential off-target effects as low as possible [21].

Therefore, the main aim of this study was to assess the in vivo short- and long-term impact of PS-IONPs in mice with persistent inflammation. First, we studied the physicochemical properties of the PS-IONPs. Later on, we established long-term inflammation in mice and used the endpoints “organ iron concentration” to obtain information on IONP-induced changes in iron organ distribution, consumption, and/or excretion. Furthermore, we studied the inflammatory status of the liver as an important metabolic organ, particularly since polysaccharides are involved in pro-inflammatory events per se. For this purpose, we determined the protein expression of members of the NF-κB signalling pathway, which play a pivotal role in the expression of pro-inflammatory genes [22]. We further determined the X-ray density of bone/bone marrow to assess the potential impact remodelling in mentioned organs. Lastly, we used the endpoints “body weight” and “haemogram” to assess and unveil signs of anaemia, and the blood plasma cytokine levels to obtain information about the immune system’s status and the body’s response.

## 2. Materials and Methods

### 2.1. Magnetic Nanoparticles

PS-IONPs were fabricated using a core-shell method. The iron oxide cores were generated by precipitating iron(II) and iron(III) sulphates under alkaline conditions. The cores (diameter lower than 10 nm) were coated with an aqueous solution of polysaccharides under high pressure homogenization. The PS-IONPs were rinsed using water for injection and collected using magnetic separation. The PS-IONPs were finally filtered (Millex glass fibre filter, Merck KGaA, Darmstadt, Germany). The hydrodynamic diameter and the zeta potential of the PS-IONPs were quantified using a Zetasizer Nano ZS (Malvern Instr. Ltd., Worcestershire, UK). The zeta potential of the PS-IONPs was determined after their suspension in a 12.5 µg/mL KCl solution in Milli-Q-water at a nanoparticle concentration of 0.5 mg/mL. Using a spectrophotometric method (Spectroquant^®^; Merck, Merck KGaA, Darmstadt, Germany), the iron concentration was assessed against an iron standard (Titrisol^®^, Merck, Merck KGaA, Darmstadt, Germany). To verify the sterility of PS-IONPs, they were cultured on agar plates and incubated for 72 h at 37 °C. All PS-IONPs were found to be sterile.

### 2.2. Animal Studies

The NMRI female mice (6 to 8 weeks of age, obtained from Janvier Labs, Saint Berthevin Cedex, France) were kept under controlled environmental conditions, including a 14/20-h light–dark cycle, a room temperature of 21 ± 2 °C, and an ambient humidity of 55 ± 5%. All animals had unrestricted access to food and water. Mice were randomly assigned to four groups, with up to nine animals per group. The experimental group, labelled as “+/+”, represented the condition where individuals with persistent inflammation were injected with PS-IONPs. The remaining groups served as controls: (1) the “−/−” group, which controlled for the noninflammatory and non-PS-IONP state, (2) the “+/−” group, which controlled for the inflammatory state only, and (3) the “−/+” group, which controlled for the PS-IONP state only (see Table 1). All procedures were performed under anaesthesia with isoflurane (2% *v*/*v* in air). The study adhered to international ethical guidelines on animal experimentation and was approved by the regional animal care committee (Thüringer Landesamt für Verbraucherschutz, Bad Langensalza, Germany, approval code: 02-027/15).

The amount of iron delivered through PS-IONPs was aligned with the recommendations from the European Medicines Agency for iron oxide–based MRI contrast agents (e.g., Sinerem^®^, Villepinte, France).

**Figure 1:**
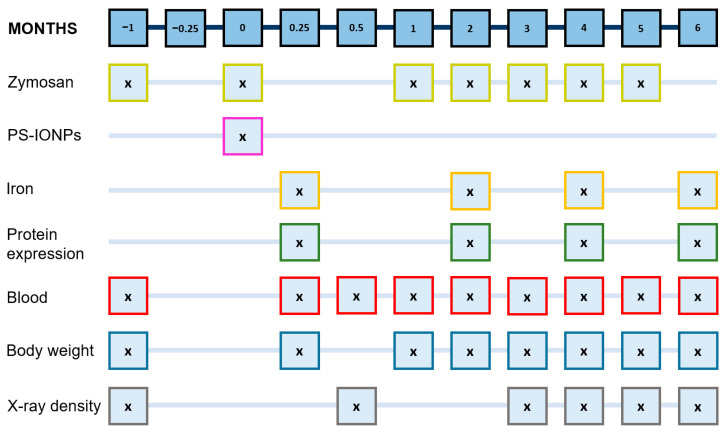
The time-dependent interventions in mice and principal endpoints during in vivo experimentation. Frequent subcutaneous injections of zymosan into the right hind leg (7 cycles of 18 mg/kg body weight each) were carried out to induce a chronic inflammatory state. PS-IONPs: intravenous injection of 50 µmol Fe/kg body weight.

The persistent systemic inflammatory state was induced using sterile zymosan (zymosan-A, Sigma-Aldrich Chemie GmbH, Steinheim, Germany), which was dissolved in sterile-filtered saline (0.9% (*w*/*v*) NaCl in water). Mice in the “+/+” and “+/−” groups received subcutaneous injections of 18 µg zymosan per kg of body weight into the right hind leg (see Table 1). This process was repeated seven times over four-week cycles. The selected zymosan dose was intentional, as higher doses can lead to excessive inflammation, which was not the objective of this study [23]. At specific post-observation intervals (see Figure 1), mice were euthanized using an overdose of isoflurane anaesthesia (5% (*v*/*v*)). The severity of local edema was assessed using a four-point scale, where “0” indicated no edema and “3” denoted edema with diameters comparable to that of a lentil. The used iron dose was calculated according to the recommendation of the European Medicines Agency for Sinerem^®^, an iron oxide–based contrast agent for MRI.

We studied different post-observation times after intravenous application of PS-IONPs: (1) 0.25 months as early post-observation time; hereby, we expected the most prominent impact of the PS-IONP application at this time-point according to previous studies on other IONP formulations (e.g., [24]); (2) a 2 to 6 month time-frame was selected to assess the long-term PS-IONP impacts associated to almost 1/10 to 2.5/10 of the total life expectancy of mice.

### 2.3. Iron Determination via Atomic Absorption Spectrometry (AAS)

Water was extracted from isolated organ tissues using a hot incubator. Next, 1 mL of a per-chloric acid (70%): nitric acid (65%) solution (volume proportions 2:3) was given to dried organs (three pieces per organ and mouse). Incineration was conducted by gradually heating the samples to 70, 160, and 250 °C, holding each temperature for 1 h, and then allowing them to cool overnight to 22 °C. Following this, 1 mL of nitric acid (1 N) was added to each tissue sample, and the iron content was subsequently measured using flame atomic absorption spectrometry (AAS 5 FL, Analytik Jena AG, Jena, Germany) with an acetylene–air flame at the analytical wavelength of 248.3 nm. Calibration for iron concentration was performed with aqueous standard solutions (0, 5, 10, 20, 30, and 50 μmol Fe per L of 0.1 N HCl; Merck KGaA, Darmstadt, Germany). A serial precision of 2.4% was determined from 21 dilutions of a single tissue sample, while day-to-day precision of 4.8% was confirmed from repeated analysis of one sample over 21 consecutive days.

### 2.4. Detection of Protein Expression via SDS-PAGE and Immunoblotting

Liver tissue samples were lysed in RIPA buffer containing protease and phosphatase inhibitors (both from Roche Diagnostics GmbH, Mannheim, Germany) and homogenized using the gentle-MACS™ Octo Dissociator (Miltenyi Biotec B.V. & Co., Bergisch Gladbach, Germany). After centrifugation, the supernatant was collected to determine the protein concentration via the Bradford assay, using BSA for calibration (absorbance: 595 nm), measured with a plate reader (Tecan Infinite M1000 Pro, Tecan Group Ltd., Männedorf, Switzerland). Sodium dodecyl sulphate polyacrylamide gel electrophoresis (SDS-PAGE, 10% (*w*/*v*) SDS gels) and Western blotting (Immobilon^®^-P, Merck Millipore Ltd., Carrigtwohill, Ireland) were used to separate and transfer proteins. The blotted membranes were blocked with PUREBlock™ (Vilber Lourmat, Deutschland GmbH, Eberhardzell, Germany) and incubated overnight at +4 °C with primary rabbit antibodies against COX-2, IκBα, Bcl-2 (1:1000; Abcam, Cambridge, UK), p100/52, p105/50 (1:1000; Cell Signaling Technology, Leiden, NL, USA), and p65 (1:1000; Santa Cruz Biotechnology, Texas, TX, USA). β-actin or GAPDH (1:10,000, Abcam) was used as the loading controls. Finally, membranes were incubated with a peroxidase-conjugated mouse anti-rabbit secondary antibody (1:10,000, Biozol Diagnostica, Eching, Germany) for 1 h at room temperature. Protein bands were detected via chemiluminescence (PURECL and Fusion FX7 Edge; Vilber Lourmat Deutschland GmbH, Eberhardzell, Germany). Quantification of protein expression was performed via densitometric analysis of protein bands using the software Bio1D (Version 15.08c, Vilber Lourmat Deutschland GmbH, Eberhardzell, Germany). Data were normalized to housekeeping protein β-actin or GAPDH (loading controls, observation times: 0.25 and 2 months vs. 2 and 4, respectively).

### 2.5. Body Weight and Haemograms

Mouse body weight was regularly monitored to evaluate the potential effects of PS-IONPs on the animals’ health. Additionally, blood samples were collected from the subclavian vein of sedated mice using 50 µL sodium-heparin–coated capillaries (Hirschmann Laborgeräte GmbH & Co. KG, Eberstadt, Germany). The collected blood was diluted 1:5 with 200 µL of isotonic saline (Fresenius Kabi AG, Bad Homburg, Germany) and analysed using an automated animal haematology analyser (Sysmex XT-1800i, Hyogo, Japan).

### 2.6. Analysis of X-Ray Density of Bones and Bone Marrow

The PS-IONP impact on bone and bone marrow remodelling was assessed by scanning mice with an in vivo mCT imager (TomoScope^®^ Synergy Twin CT Imaging, Erlangen, Germany) using a standard protocol with an X-ray dose of 65 kV. Anatomical pictures were gained using the Imalytics software (Version: Preclinical 2.1, Gremse-IT GmbH, Aachen, Germany). The X-ray absorption intensity (Hounsfield units) of bone marrow and bones were determined using regions of interest (ROI; bones: *n* = 4 ROIs á 0.2 mm^2^ each, 37 pixels; bone marrow: one ROI from 0.62 to 5.21 mm^2^ depending on the CT-slice intersection plane, between 116 and 977 pixels).

### 2.7. Analysis of Mediator Secretion in Blood Plasma

Blood was collected in lithium-heparin tubes containing a separating gel (KABE LABORTECHNIK GmbH, Nümbrecht-Elsenroth, Germany). After centrifugation of the blood sample (5 min, 5000 rcf), the plasma layer was collected and analysed using multiplexed ELISA (LEGEND-plex™ Mouse Inflammation Panel, BioLegend, San Diego, CA, USA) according to the instructions of the manufacturer.

### 2.8. Statistical Analysis

Data were presented as mean ± standard deviation. Statistical differences between the animal groups were considered significant, with corresponding *p*-values reported in the text. To unveil long-time dependent effects (organ iron distribution, body weight, and blood parameters), we plotted the data in dependence on time (0.25 to 6 months) and used linear regression analysis with confidence intervals of 95%. Regression lines showing coefficient slopes significantly different from 0 with *p* < 0.05 were depicted as red lines in the corresponding graphs.

## 3. Results

The physicochemical analysis of the PS-IONPs revealed hydrodynamic diameters, which were among the larger ones, among those frequently used in research and development activities, given by their good circulation half-life and reduced hepatic filtration compared to smaller nanoparticle sizes [25]. Due to the polysaccharide coating containing D-glucuronic acid with carboxylic end-groups, the zeta-potential of the IONPs was negative. The low polydispersity-index indicate a quite narrow particle distribution (see also Supplementary Figure S1). Consequently, PS-IONPs are uniform in size, which assures controlled biological interactions as well as consistent and reliable experimental outcomes (Table 2). The nanoparticle core diameter and the presence of surface functional groups were similar to those reported by other studies (e.g., [26,27,28]).

**Figure 2:**
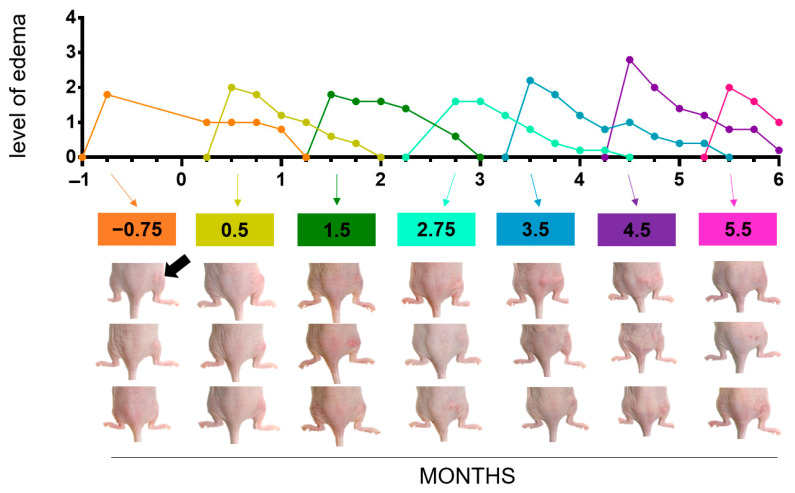
Level of edema as a result of subcutaneous injection of zymosan into mice of the “+/+” animal group. As a component of the cell wall from *Saccharomyces cerevisiae*, zymosan is known to activate various immune cells, which release cellular mediators promoting vascular permeability. Arrow: exemplary anatomical location of local edema at their right hind legs. The animal pictures refer to different time-points (in months, highlighted as coloured boxes) and are representative for all animals of the group. For clarity reasons, we do not depict the standard deviations of the mean in the graph.

Upon induction of the persistent inflammatory state by subcutaneous injection of zymosan (for details see Figure 1, Table 1, and Section 2), all mice morphologically showed local edema at their right hind legs. This is attributed to local exudation of protein-rich fluid, vasodilatation, and cell migration to the injured site as a result of the activation of resident macrophages and mast cells [29] (Figure 2 and Supplementary Figure S2).

**Figure 3:**
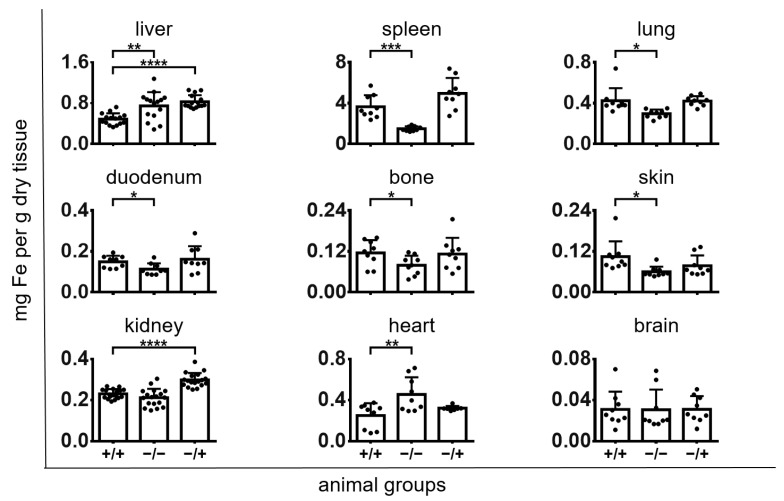
Short-term effects of PS-IONPs on the iron distribution in the organs of mice with a persistent inflammatory state in comparison to the control groups (0.25 months after intravenous application). Experimental group: “+/+”—animals with a persistent inflammatory state (zymosan: 7 cycles of 18 µg/kg body weight, PS-IONPs: 50 µmol Fe/kg body weight). Control groups: “−/−”—animals without persistent inflammation and no intravenous PS-IONP injection, and “−/+”—animals without persistent inflammation but with intravenous injection of PS-IONPs. Data are represented as iron content in milligrams per gram of dry tissue mass (individual data points), along with the mean and standard deviation; * *p* < 0.05, ** *p* < 0.01, *** *p* < 0.001, **** *p* < 0.0001 (*t*-test with Welch’s correction).

After a single application of PS-IONPs at a dose according to the recommendation of the European Medicines Agency for Sinerem^®^ (see Section 2), the elucidation of the short-term effects of PS-IONPs to inflammatory stressed mice (animal group “+/+”) showed distinct relationships regarding the iron distribution in organs. This particularly applies when comparing the iron concentration from animals of the group “+/+”on one side versus groups “−/−” and “−/+” on the other side (0.25 months, see also Figure 3 and Table 1). In this context, the presence of PS-IONPs decreased the iron levels of the liver and increased the iron levels of the spleen, lung, duodenum, and skin of inflammatory stressed mice. In the kidney, the administration of PS-IONPs attenuated the iron accumulation of the “+/+” animal group (compare with groups “−/−” and “−/+”). In the heart of the “+/+” animal group, the iron level was decreased, whereas no distinct effects were observed for the brain in the shortterm.

**Figure 4:**
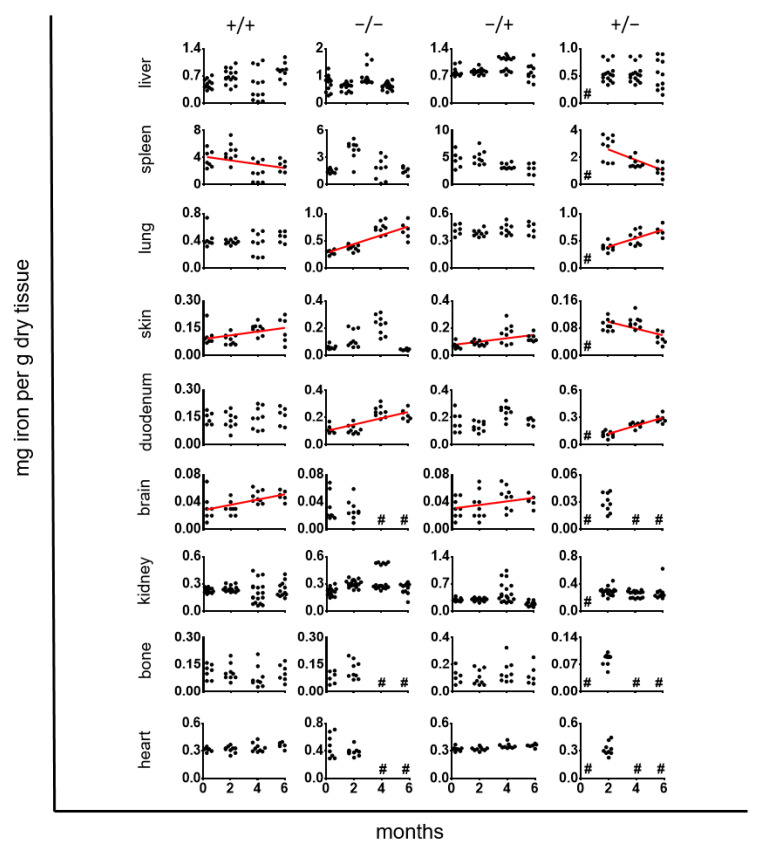
Long-term impact of PS-IONPs on the iron distribution in the organs of mice with persistent inflammatory and intravenously injected PS-IONPs in comparison to the control groups. Experimental group: “+/+”—animals with a persistent inflammatory state (zymosan: 7 cycles of 18 µg/kg body weight, PS-IONPs: 50 µmol Fe/kg body weight). Control groups: “−/−”—animals without persistent inflammation and no intravenous PS-IONP injection, and “−/+”—animals without persistent inflammation but with intravenous injection of PS-IONPs, “+/−”—animals with persistent inflammation but without intravenous injection of PS-IONPs. Data are represented as iron content in milligrams per gram of dry tissue mass (individual data points). Only regression lines with R2 larger than 0.12 and with slopes significantly nonzero with *p* < 0.05 were depicted (red lines). # not determined.

The long-term impact of PS-IONPs on the organ iron concentration of inflammatory stressed mice (“+/+” animal group) showed different contributions, which were related to the presence of IONPs (group “−/+”) and/or the inflammatory state (group “+/−”; up to 6 months upon intravenous PS-IONP injection, Figure 4). In this view, the liver revealed no distinct specific time-dependent variations of the organ iron concentration. The iron levels of the spleen decreased with time, which can mostly be attributed to the inflammatory state. Moreover, the administration of PS-IONPs attenuated the normal age-dependent increase in iron levels in the lung (comparison of group “+/+” with groups “−/−” and “−/+”). Finally, the administration of the PS-IONPs to the “+/+” animal group abolished the normal age-dependent increase in the iron concentration in the duodenum (compare group “+/+” with the “−/−” animal group), but it increased the iron accumulation in the skin. No distinct effects were observed in relation to the kidneys, bones, and heart.

**Figure 5:**
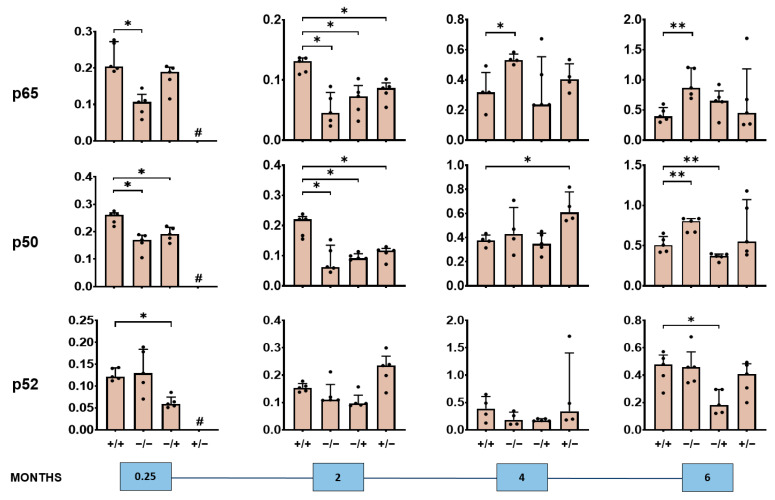
Long-term impact of expression of PS-IONPs on NF-κB nuclear factor protein expression in the liver of mice with a persistent inflammatory state in comparison to the control groups. Experimental group: “+/+”—animals with a persistent inflammatory state (zymosan: 7 cycles of 18 µg/kg body weight, PS-IONPs: 50 µmol Fe/kg body weight). Control groups: “−/−”—animals without persistent inflammation and no intravenous PS-IONP injection, and “−/+”—animals without persistent inflammation but with intravenous injection of PS-IONPs, “+/−”—animals with persistent inflammation but without intravenous injection of PS-IONPs. Protein expression is presented relative to the housekeeping proteins β-actin or GAPDH (dots) and as mean and standard deviation of the mean; * *p* < 0.05, ** *p* < 0.01 (Mann–Whitney U-test), # not determined.

**Figure 6:**
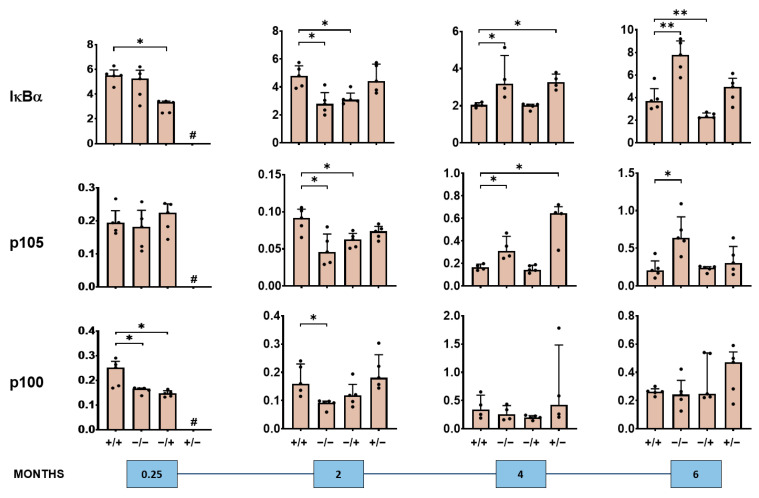
Long-term impact of PS-IONPs on NF-κB regulator protein expression in the liver of mice with a persistent inflammatory state in comparison to the control groups Experimental group: “+/+”—animals with a persistent inflammatory state (zymosan: 7 cycles of 18 µg/kg body weight, PS-IONPs: 50 µmol Fe/kg body weight). Control groups: “−/−”—animals without persistent inflammation and no intravenous PS-IONP injection, and “−/+”—animals without persistent inflammation but with intravenous injection of PS-IONPs, “+/−”—animals with persistent inflammation but without intravenous injection of PS-IONPs. Protein expression is presented relative to the housekeeping proteins β-actin or GAPDH (dots) and as mean and standard deviation of the mean; * *p* < 0.05, ** *p* < 0.01 (Mann–Whitney U-test), # not determined.

**Figure 7:**
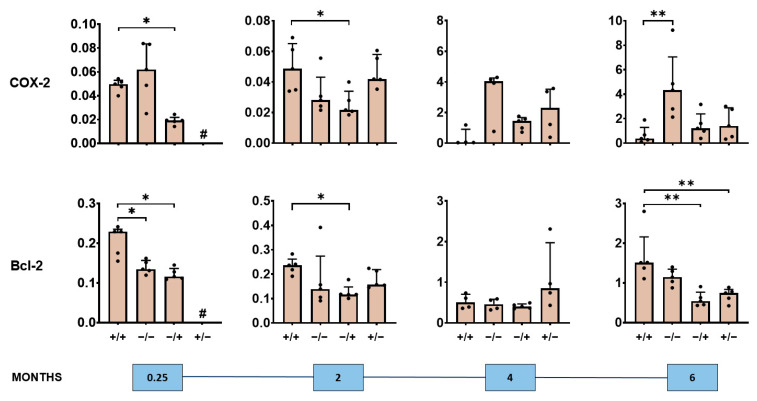
Long-term impact of PS-IONPs on NF-κB downstream effector protein expression in the liver of mice with a persistent inflammatory state in comparison to the control groups. Experimental group: “+/+”—animals with a persistent inflammatory state (zymosan: 7 cycles of 18 µg/kg body weight, PS-IONPs: 50 µmol Fe/kg body weight). Control groups: “−/−”—animals without persistent inflammation and no intravenous PS-IONP injection, and “−/+”—animals without persistent inflammation but with intravenous injection of PS-IONPs, “+/−”—animals with persistent inflammation but without intravenous injection of PS-IONPs. Protein expression is presented relative to the housekeeping proteins β-actin or GAPDH (dots) and as mean and standard deviation of the mean; * *p* < 0.05, ** *p* < 0.01 (Mann–Whitney U-test), # not determined.

We also observed a short- and long-term impact of the PS-IONPs on the expression of NF-κB–associated proteins in the liver of inflammatory stressed mice (Figure 5, Figure 6 and Figure 7, Supplementary Figure S3). In particular, there was an overexpression of the NF-κB nuclear factors p65 and p50 detectable, but not of p52 in the short term (0.25 and 2 months post-observation time), and signs of attenuated expression of said proteins in the long term (4 and 6 months post-observation time; compare groups “+/+” with “−/−” in Figure 5). Such effects seem to be related to the presence of the PS-IONPs and/or the low-grade inflammation state. Beyond this, the expression of the NF-κB regulator proteins IκBa, p105, and p100 in the liver of inflammatory stressed and PS-IONP-applied animals was increased in the short term but attenuated at longer post-observation times (particularly IκBα and p105; compare group “+/+” with “−/−” in Figure 6). The same applies for the NF-κB down-stream proteins COX-2 and Bcl-2 in the liver of inflammatory stressed and PS-IONP–applied mice, which showed an increased expression at the short term and an attenuated one at the long term (compare group “+/+” with “−/−” in Figure 7).

**Figure 8:**
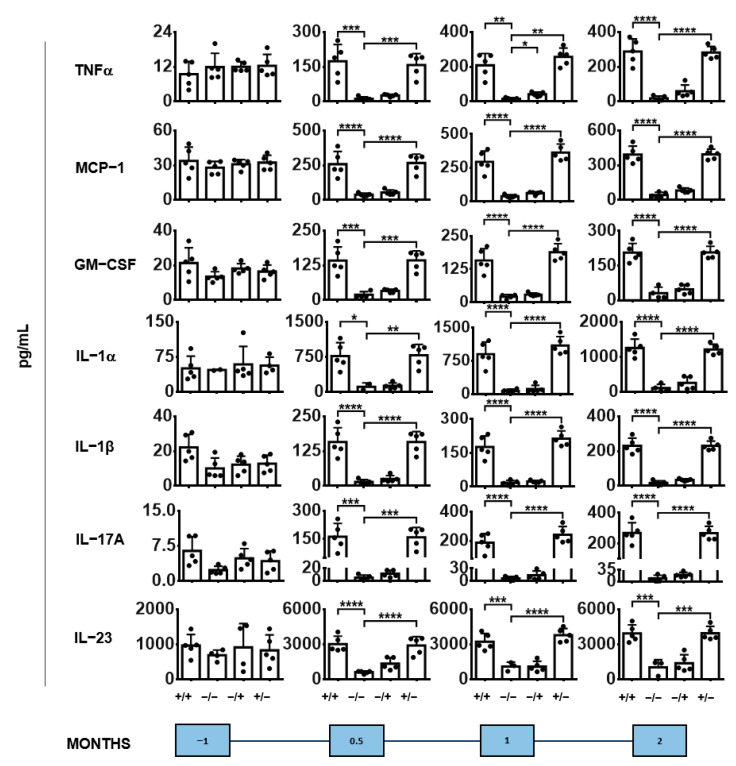
Cytokine levels of blood plasma with pro-inflammatory potential. Experimental group: “+/+”—animals with a persistent inflammatory state (zymosan: 7 cycles of 18 µg/kg body weight, PS-IONPs: 50 µmol Fe/kg body weight). Control groups: “−/−”—animals without persistent inflammation and no intravenous PS-IONP injection, and “−/+”—animals without persistent inflammation but with intravenous injection of PS-IONPs, “+/−”—animals with persistent inflammation but without intravenous injection of PS-IONPs. Data are plotted as mean and standard deviation of the mean of 3 to 5 animals per group, * *p* < 0.05, ** *p* < 0.01, *** *p* < 0.001, **** *p* < 0.0001 (one-way ANOVA with Tukey’s multiple comparisons test).

Interestingly, the administration of PS-IONPs to inflammatory stressed mice attenuated signs of inflammatory-based anaemia as the haemograms show (Supplementary Figure S4). In particular, they attenuated the natural and age-dependent decrease in the red blood cell count (RBC; −/− animal group), haemoglobin content (HGB; +/− animal group), and haematocrit (HCT; −/− and +/− animal group), but they specifically reduced the main corpuscular haemoglobin (MCH; +/+ animal group) and the mean corpuscular haemoglobin concentration (MCHC; +/+ animal group see Supplementary Figure S4) with increasing observation times after nanoparticle injection. The impact of the PS-IONPs on the long-term dynamics of cytokine secretion to the blood of inflammatory stressed mice was much lower than that resulting from the inflammatory state by zymosan injection (Figure 8). A similar situation applies for cytokines with anti-inflammatory potential (Supplementary Figure S5). Additionally, the presence of the PS-IONPs in inflammatory stressed mice had no long-term effect on the body weight dynamics (Supplementary Figure S6). Finally, they induced a slight increase in the bone marrow density, but they had no effects on bone destruction and/or remodelling as determined via in vivo mCT imaging (Supplementary Figures S7 and S8).

## 4. Discussion

Our data show that PS-IONPs induced several short-term effects on inflammatory stressed mice as revealed by the modified iron levels of distinct organs and NF-κB protein expression profiles in liver tissue. In the long term, they modified the age-dependent iron accumulation in the lung and duodenum, but they increased the iron accumulation in the skin of inflammatory stressed animals. In the long term, there was an attenuated expression of several NF-κB–related proteins as well as attenuated inflammatory-based anaemia detectable in blood plasma. Finally, PS-IONPs had weak effects on cytokine levels of blood plasma compared to those related to the persistent inflammatory state of the animals, which was induced via zymosan injection.

The PS-IONPs used in this study are considered as nanocomposites [30]. They exhibited a surface charge, surface functional groups, and hydrodynamic diameters, which were similar to those studied by Rabel et al. [27] after suspension in simulated biological fluids, such as plasma and lysosomal fluid according to Marques et al. [31]. Due to the comparable physicochemical features of the IONPs reported by Rabel et al. with those of our PS-IONPs, we expect that PS-IONPs aggregated after exposure to body fluids (pH 7.4, 37 °C), whereas the shell was stable. According to the observations of Rabel et al., we further expect that our PS-IONPs degrade very slowly in an endolysosomal environment (artificial lysosomal medium, pH 4.5) with a complete iron release after approximately 29 days of incubation. According to this, the PS-IONP degradation/metabolization in endolysosomes should have been an ongoing process at our early post-observation time of 0.25 months and completed before the second one (2 months). It is of note that the data published by Rabel et al. was compiled in the frame of the same joint research project as the present study (nanoBEL, German Federal Ministry of Education and Research).

The core size of the PS-IONPs was between 10 and 40 nm, which is optimal for superparamagnetic behaviour (strong T2 contrast in MRI, good magnetic targeting potential). Furthermore, the PS-IONPs’ hydrodynamic size can be classified as quite large when considering the typical nm frame, which has been suggested for biomedical applications. This frame is between 20 and 100 nm for appropriate nanoparticle circulation time, stability, and efficient drug delivery. The reason for the quite large hydrodynamic size of our PS-IONPs is an increased polymer layer thickness to avoid magnetic dipole–dipole interactions. In fact, as a consequence of their high magnetic attraction, IONP crystals highly tend to clump, giving large clusters. Therefore, high polymer layer thickness helps stabilize the IONPs in suspension, ensuring that they maintain their single-domain structure and retain superparamagnetism [32].

We administered only one PS-IONP dose of 50 µg iron/kg body weight. This dose is comparable with those recommended for the use of iron-based contrast agents for MRI (e.g., for the assessment of liver and cardiac conditions, e.g., [33,34], 10 to 125 µmol iron/kg according to corresponding data sheets of IONPs for MRI). In relation to potential applications for drug release, the used iron dose would prospectively ensure an effective IONP biodistribution and allow for controlled drug release or magnetic targeting. For IONP-based drug delivery purposes, iron concentrations between 10 and 100 µmol Fe/kg have been used so far, with the specific concentration depending on the desired balance between the magnetic properties, IONP circulation time, drug loading capacity etc. (e.g., [35,36]).

The observed short-term effects of PS-IONPs on inflammatory stressed mice are well in line with the degradation dynamics in the in vitro situation published by Rabel et al. Such effects are the result of the nanoparticle clearance from circulation by highly phagocytosing macrophages, particularly in the liver, spleen, lung, and other organs. In the liver, they represent 30 to 35% of total cells [37]. Due to their anatomical location at the sinusoidal periportal regions of the liver, Kupffer cells have very good accessibility to particulates in general [38,39].

We attribute the short-term decrease in iron levels in the liver to an increased demand of iron as a consequence of activated Kupffer cells in inflammatory stressed animals. In this view, important pro-inflammatory stressors are (1) the presence of zymosan molecules as “pseudo-pathogens” [22] and (2) iron-based oxidative stress [40] from the metabolization of internalized PS-IONPs. Additionally there is a tight interrelation of Kupffer cells with other liver cells (e.g., hepatocytes, stellate cells, endothelial cells, etc. [41]). Consequently, these highly stressed Kupffer cells could have influenced specific biochemical processes in hepatocytes (e.g., on the activity of cytochrome P450 [42]). Stressed Kupffer cells are further able to activate various metabolic processes in immune cells [43,44]. Such intensive cell-to-cell interactions very well explain the high demand of iron by the liver [45]. Finally, the same iron demand could have further fostered the iron redistribution in the spleen, lung, skin, and bones of the “+/+” animal group.

Additionally, in the short term, the high demand of iron by the liver of “+/+” animals seems to be intensified by the presence of polysaccharides on the IONP’s surface, as our results show. This deduction is reinforced by the fact that when administering IONPs coated with PEI to inflammatory stressed mice, there is no such a decreased iron concentration detectable in the liver [24]. These findings strongly indicate the influence of pro-inflammatory stimuli induced by the introduction of polysaccharides from the IONPs. In fact upon uptake of polysaccharides, Kupffer cells can differentiate into pro-inflammatory (M1-like) macrophages, which are known to produce and secrete a variety of pro-inflammatory cytokines, including tumour necrosis factor-alpha (TNF-α), interleukin-1 (IL-1), and interleukin-6 (IL-6) [37]. The release of these cytokines plays a crucial role in orchestrating the immune response and can lead to inflammation in the liver and systemic circulation [37]. The pro-inflammatory cytokines released by Kupffer cells were shown to contribute to the development of liver inflammation and are implicated in various liver diseases [46,47].

In the long term and in the lung and duodenum of inflammatory stressed animals, the presence of the PS-IONPs seem to attenuate the normal time-dependent accumulation of iron. Possibly, the polysaccharides introduced from nanoparticle degradation may have influenced iron levels particularly for the production of important cellular mediators [48] in mentioned organs. In fact, cellular mediator expression requires iron from well-known iron store proteins called “ferritins”, which consist of multimeric spheres forming proteins filled up with 4500 iron atoms each [49]. Additionally, our observations may also be seen as the result of the body’s own regulation of iron homeostasis, i.e., a balance between iron absorption, utilization, and recycling in a persistent systemic inflammatory context [50]. This balance is crucial because both iron deficiency and iron overload can have detrimental effects on health. On the other hand, the long-term decrease in iron in the duodenum of “+/+” animals can be allocated to an altered regulation of iron absorption [50] in response to a long-term inflammatory state.

In the skin of “+/+” animals, we found a continuous long-term accumulation of iron. The underlying mechanisms are unclear, since cutaneous deposition of iron has been occasionally described [51]. Therefore, further investigations are needed on this subject. Moreover, the long-term accumulation of iron in the brain of inflammatory stressed mice administered with PS-IONPs may very well be the result of a compromised blood–brain barrier [52] together with the facilitated uptake of the IONPs by astrocytes, which was mediated by the polysaccharide coating [53]. Additionally, transferrin-mediated uptake in brain endothelial cells is highly probable, because of overexpression of transferrin receptors in those cells [54]. Finally, we relate the long-term decrease in iron in the spleen of “+/+” animals with the fact that the spleen is an important retention site of iron from macrophages [55], as well as to the high iron demand during the long-lasting inflammatory state.

With consideration of the expression of NF-κB associated proteins in the liver of inflammatory- and PS-IONP-stressed animals, the increased short-term expression of the nuclear factors p65 and p50 is well in agreement with the pro-inflammatory context of the liver elucidated above, as well as with the high blood plasma levels of pro-inflammatory cytokines. In this pro-inflammatory cellular context, the introduction of additional sugar molecules by degradation of the IONP coating could have favoured the activation of NF-κB–signalling pathways [56] in Kupffer cells. Additionally, a pro-inflammatory cellular context similar to that explained for Kupffer cells is also expected in relation to hepatocytes [57], which account for 60 to80% of the total cell population and volume of the liver. In this view, NF-κB is needed to regulate the expression of genes involved in glucose and lipid metabolism, oxidative stress, hepatocyte proliferation and survival [38,41]. Therefore, and in very unfavourable cases, repeated short-term administration of PS-IONPs may lead to a persistent activation of NF-κB and, finally, to metabolic dysfunction of the liver in the clinical situation.

Furthermore, we relate the short-term impact of PS-IONPs on the expression of NF-κB–associated proteins in the liver of inflammatory stressed animals, at least in parts, to ferroptosis in target cells (e.g., in Kupffer cells) as result of iron overload, which is an iron-dependent form of cell death. Herein, inflammatory signalling pathways which include NF-κB [58,59] and its downstream proteins COX and Bcl-2 are known to be involved [60,61].

Whereas the short-term expression of NK-κB–associated proteins in the liver of the “+/+” animal group exhibited pro-inflammatory features, it turned into an anti-inflammatory behaviour in the long term. We suggest that the liver returned to immunological homeostasis [62] after having finalized degrading and metabolizing the PS-IONP, which sequentially reduced corresponding pro-inflammatory stimuli in the mentioned organ.

Finally, the administration of PS-IONPs attenuated the signs of inflammatory-based anaemia in inflammatory stressed mice in the long term. It is already well known that iron is a critical component of haemoglobin present in red blood cells. The inflammatory environment does further impair normal red blood cell production, leading to anaemia [13]. In our study, the systemic availability of iron after IONP degradation may well have alleviated inflammation-mediated anaemia and increased the incorporation of iron in haematopoietic stem cells of the bone marrow [63]. The reasons for a concomitantly decreased MCH and MCHC are unknown; researchers should clarify the corresponding reasons in the future. Beyond this, PS-IONPs per se had weak effects on cytokine release into the blood as opposed to those resulting from the inflammatory state of the animals. Presumably, PS-IONPs induce pro-inflammatory cytokine release very early after intravenous administration and therefore transitorily at time-points earlier than that used in this study (0.25 months). Therefore, further investigations should shed more light into this issue.

**Figure 9:**
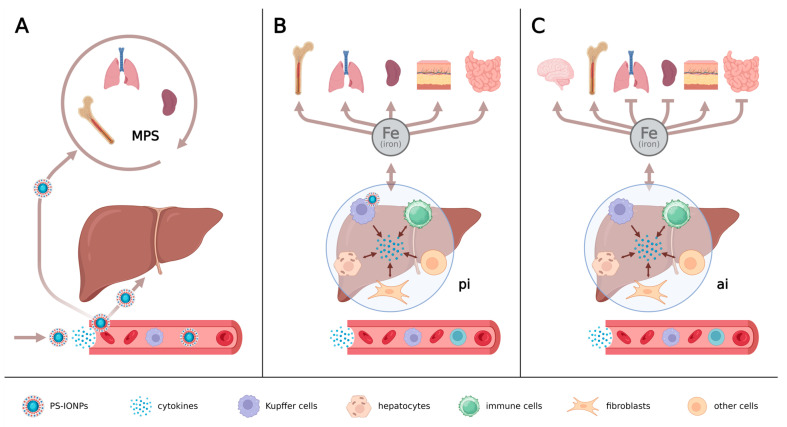
Simplified organ map showing the systemic influence of PS-IONPs in animals with persistent inflammation. (**A**) Immediately after intravenous injection of PS-IONPs, (**B**) short-term situation (2 to 4 months thereafter), and (**C**) long-term situation (4 to 6 months thereafter). pi: pro-inflammatory impact of PS-IONPs in the liver after extensive accumulation in Kupffer cells, ai: anti-inflammatory impact of PS-IONPs in the liver, MPS: organs of the mononuclear phagocyte system. Created with BioRender.com.

Our data show that when PS-IONPs are administered to mice with persistent inflammation at doses relevant for MRI (50 µg iron/kg body weight), they will not immediately overwhelm the body’s regulatory systems (Figure 9). Nevertheless, the mentioned iron dose was much higher than the body’s daily iron absorption. Therefore, repeated applications of IONPs at equivalent iron doses should be performed at time intervals longer than 2 months in order to prevent excessive organ damage of inflammatory-stressed individuals. In such cases, the pathophysiological parameters of the liver, spleen and other organs need to be carefully monitored and controlled. Finally, the benefits of high iron doses from IONPs must be weighed against these risks.

In the view of the translation of our data to the clinical situation, potential limitations are that we considered only one persistent animal inflammation model and only one organ of the MPS (liver). Therefore, further detailed investigations should be performed in the future.

## 5. Conclusions

Taking all data together, the intravenous injection of PS-IONPs in individuals with persistent inflammation induces a short- and long-term redistribution of iron in organs, which is associated with an increased iron demand, since iron is involved in many pro-inflammatory events. It further induces a transitory pro-inflammatory impact on the metabolism of the liver, which is the result of the incorporation of free sugar molecules and iron from nanoparticle clearing, metabolization, and degradation. In the long-term, PS-IONPs have the potential to attenuate the NF-κB–related pro-inflammatory status of the liver and to attenuate inflammation-related anaemia. PS-IONPs do not persistently affect the immune system status of inflammatory-stressed individuals, as revealed by the cytokine levels in blood plasma. In general, our PS-IONPs are biocompatible. Nevertheless, and due to their short-term pro-inflammatory impact, the administration of PS-IONPs should prospectively be applied with caution in patients with inflammatory diseases of the liver, such as hepatitis or metabolic disorders, since NF-κB signalling is required for inflammatory processes as well as for several other liver cell functions. Finally, our data does not only help in assessing the safety and efficacy of IONPs, but also contributes to the development of personalized and optimized nanoparticle-based therapies in general.

## Figures and Tables

**Table 1:** Experimental (E) and control (C) mice groups of this study. Animal groups “+/+” and “−/+” received PS-IONPs (50 µmol iron/kg body weight, 64% (*w*/*w*) iron, experimental day 0, see Figure 1) via the tail vein. The persistent systemic inflammation state was induced by regular subcutaneous injection of zymosan into mice (for details see Section 2).

Animal Group Name	Systemic Inflammation	Injected PS-IONP	Description
+/+	Yes	Yes	E
−/−	No	No	C
−/+	No	Yes	C
+/−	Yes	No	C

**Table 2:** Physicochemical features of the PS-IONPs of the present study. PDI: polydispersity-index, size of the core < 10 nm.

Z-Average * (nm)	Hydrodynamic Diameter (nm) **	Zeta Potential (mV)	PDI
135 ± 35	90	−36 ± 0.9	0.104 ± 0.0

Weighted mean hydrodynamic diameters: * by light intensity and ** by nanoparticle number.

## Data Availability

The raw datasets supporting the conclusions of this article will be made available by the authors on request.

## Supplementary Materials

The following supporting information can be downloaded at: https://www.mdpi.com/article/10.3390/jox14040091/s1, Figure S1: Hydrodynamic size distribution of PS-IONPs weighted by nanoparticle number as determined by dynamic size scattering (Zeta-Sizer from Malvern); Figure S2: Level of edema as result of subcutaneous injection of zymosan into mice with persistent inflammation (“+/−” animal group); Figure S3: Exemplary pictures showing the protein bands gained via SDS-PAGE and Western/immunoblotting of liver lysates from animals after 6 months of intravenous application of PS-IONPs; Figure S4: Haemogram of animals with low-grade persistent inflammatory state and intravenously administered PS-IONPs in comparison to control groups; Figure S5: Cytokine levels of blood plasma with anti-inflammatory potential; Figure S6: Body weight of mice with low-grade persistent inflammatory and intravenously injected PS-IONPs in comparison to control groups; Figure S7: X-ray density of bone marrow and bones of animals with persistent inflammatory and intravenously injected PS-IONPs in comparison to control groups; Figure S8: Typical CT pictures of different mouse body planes obtained from in vivo mCT imaging (TomoScope^®^ Synergy Twin) using a standard protocol with an X-ray dose of 65 kV; Figure S9: Exemplary unadjusted and uncropped pictures of immunoblotting membranes showing the same protein bands from Supplementary Figure S3, which were gained from liver lysates from animals after 6 months of intravenous application of PS-IONPs.
