# Supplementary material for: The Long-Term Impact of Polysaccharide-Coated Iron Oxide Nanoparticles on Inflammatory-Stressed Mice
Source: J Xenobiot. 2024 Nov 7;14(4):1711–28. doi: 10.3390/jox14040091 (PMC11587046; doi:10.3390/jox14040091)
Supplement: Supplementary file 1 [file jox-14-00091-s001.zip › jox-3170671-supplementary.pdf]

Journal of
Xenobiotics

Supplementary Materials: The Long-Term Impact of Polysaccharide-Coated Iron Oxide Nanoparticles on Inflammatory-Stressed Mice

Julia Göring, Claudia Schwarz, Eric Unger, Rainer Quaas, Ingrid Hilger

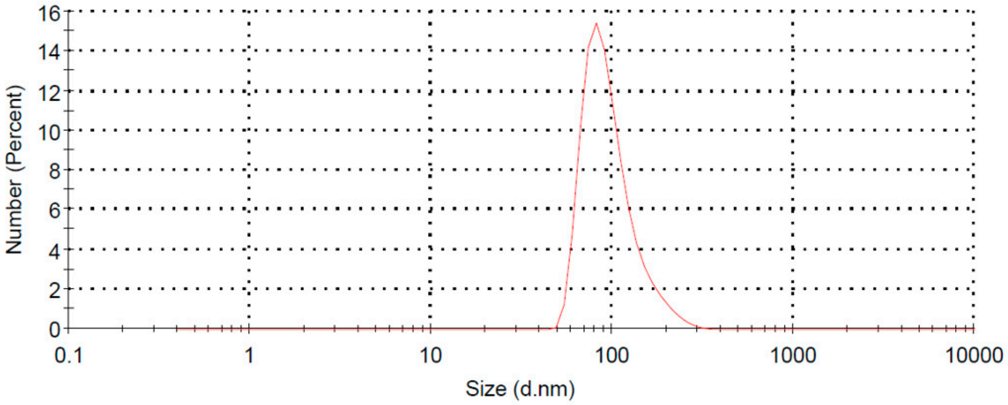

Supplementary Figure S1. Hydrodynamic size distribution of PS-IONPs weighted by nanoparticle number as determined by dynamic light scattering (Zeta-Sizer from Malvern)

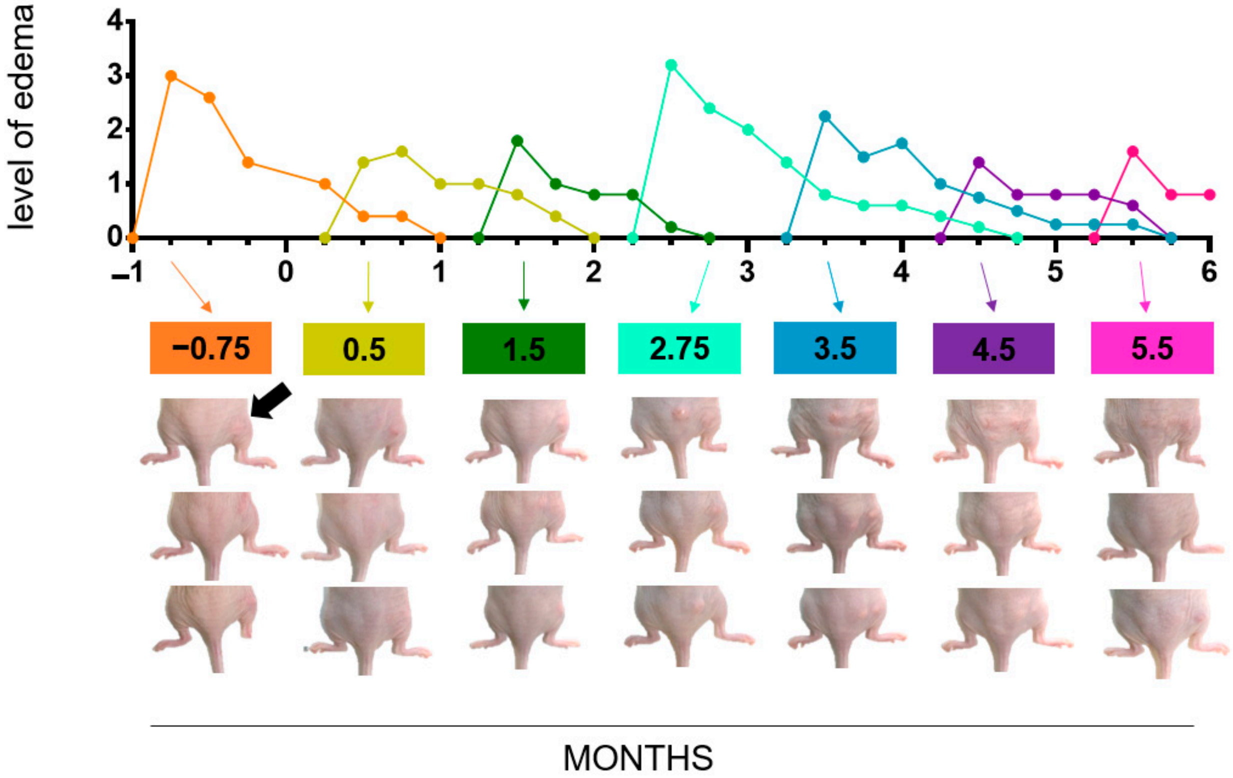

Supplementary Figure S2. Level of edema as result of subcutaneous injection of zymosan into mice with persistent inflammation ("+/−" animal group). As component of the cell wall from Saccharomyces cerevisiae, zymosan is known to activate various immune cells, which release cellular mediators promoting vascular permeability. Arrow: exemplary anatomical location of local edema at their right hind legs. The animal pictures refer to different time-points (in months, highlighted as colored boxes) and are representative for all animals of the group. For clarity reasons, we do not depict the standard deviations of the mean in the graph.

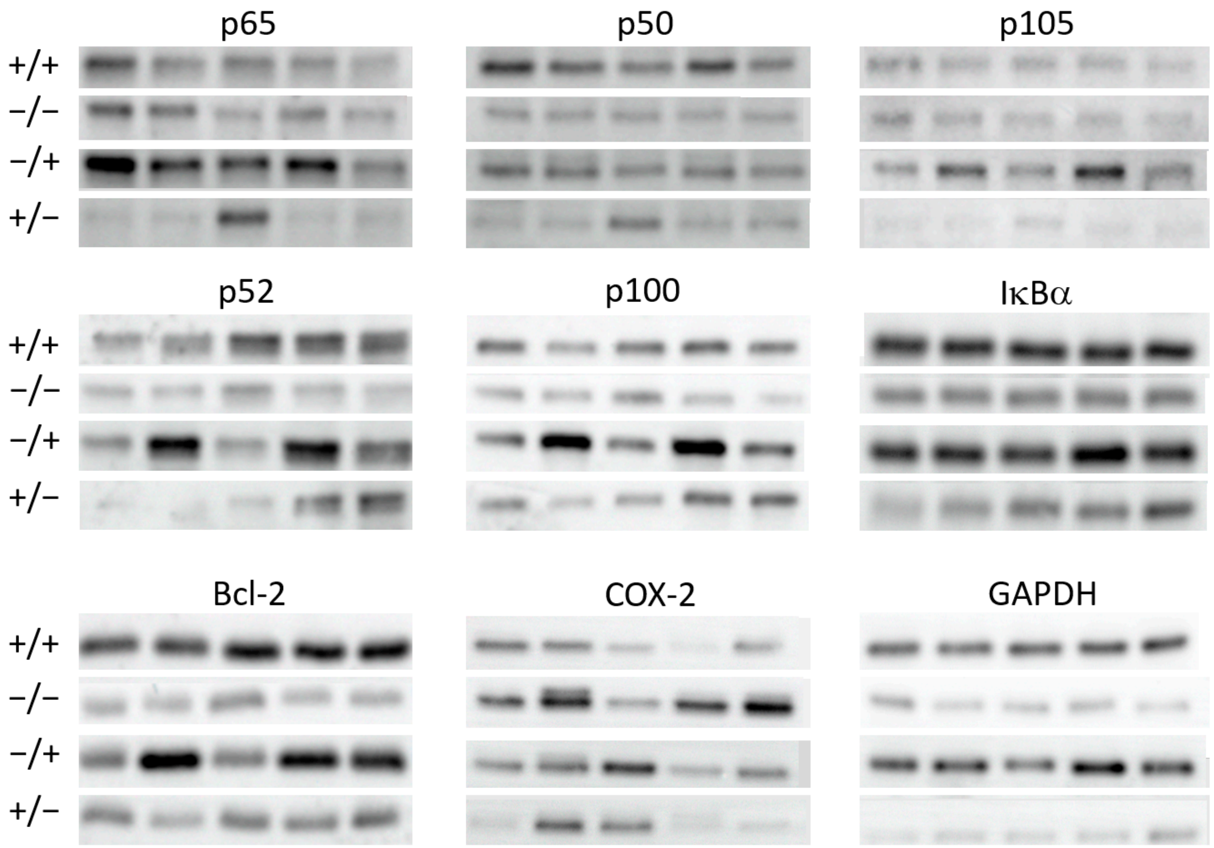

Supplementary Figure S3. Exemplary pictures showing the protein bands gained via SDS-PAGE and Western/immunoblotting of liver lysates from animals after 6 months of intravenous application of PS-IONPs. Experimental group: "+/+": animals with low-grade persistent inflammation state (zymosan: 7 cycles á 18 µg/kg body weight, PS-IONPs: 50 µmol Fe/kg body weight). Control groups: "−/−": animals without low-grade persistent inflammation state and no intravenous injection with PS-IONPs, "−/+": animals without low-grade persistent inflammation state but with intravenous injection of PS-IONPs, "+/ −": animals with persistent inflammation but without intravenous injection of PS-IONPs.

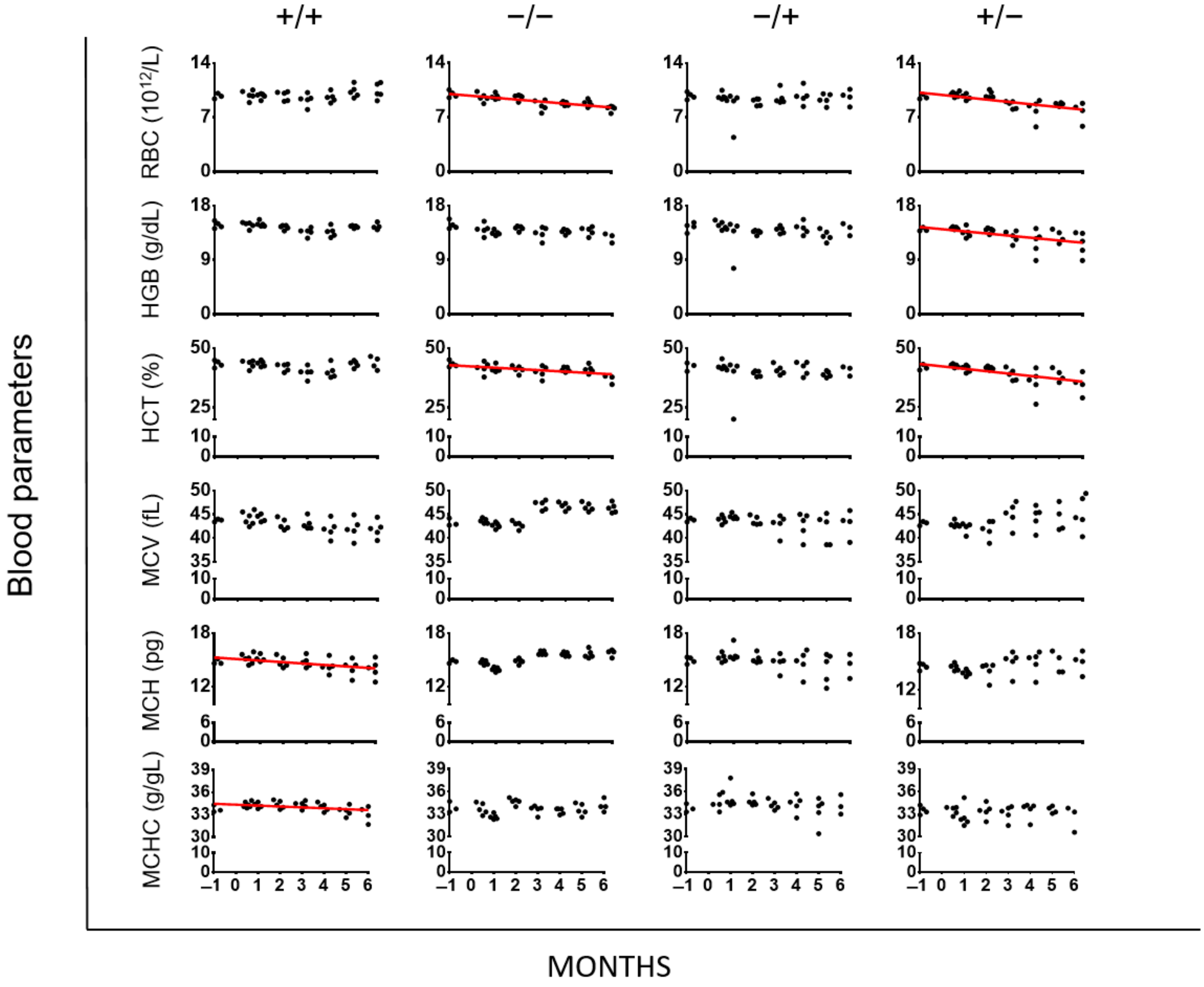

Supplementary Figure S4. Hemogram of animals with low-grade persistent inflammatory state and intravenously administered PS-IONPs in comparison to control groups. Experimental group: "+/+": animals with low-grade persistent inflammation state (zymosan: 7 cycles á 18 µg/kg body weight, PS-IONPs: 50 µmol Fe/kg body weight). Control groups: "−/−": animals without low-grade persistent inflammation state and no intravenous injection with PS-IONPs, "−/+": animals without low-grade persistent inflammation state but with intravenous injection of PS-IONPs, "+/ −": animals with persistent inflammation but without intravenous injection of PS-IONPs. Data are plotted as mg iron per g dry tissue mass of 3 to 5 animals per group. Only regression lines with R2 larger than 0.12 and with slopes significantly non-zero with $p < 0.05$ were depicted (red lines).

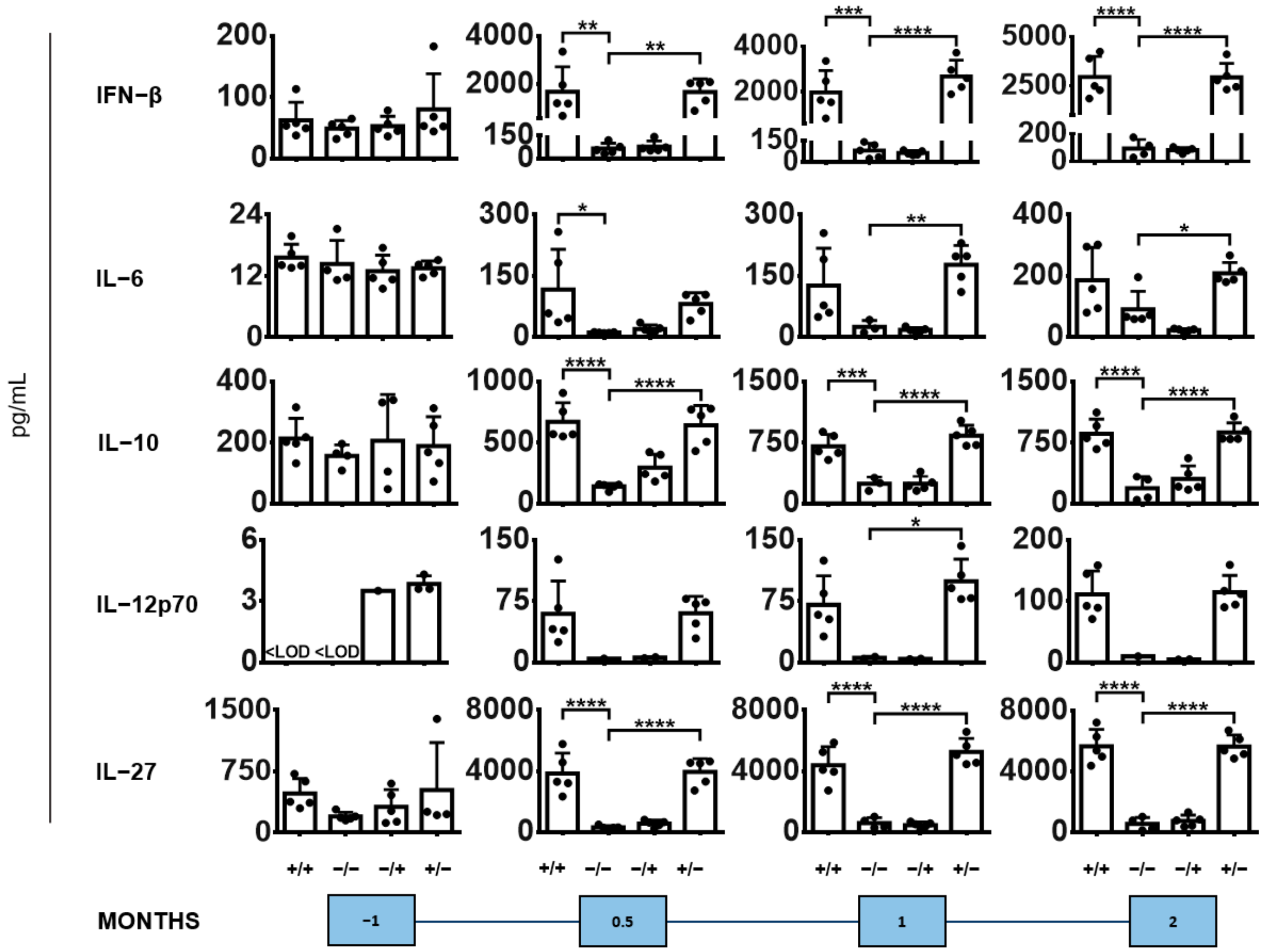

Supplementary Figure S5. Cytokine levels of blood plasma with anti-inflammatory potential. Experimental group: "+/+": animals with low-grade persistent inflammation state (zymosan: 7 cycles á 18 µg/kg body weight, PS-IONPs: 50 µmol Fe/kg body weight). Control groups: "−/−": animals without low-grade persistent inflammation state and no intravenous injection with PS-IONPs, "−/+": animals without low-grade persistent inflammation state but with intravenous injection of PS-IONPs, "+/ −": animals with persistent inflammation but without intravenous injection of PS-IONPs. Data are plotted as mean and standard deviation of the mean of n = 3 to 5 animals per group, * $p < 0.05$, ** $p < 0.01$, *** $p < 0.001$, **** $p < 0.0001$ (one-way ANOVA with Tukey's multiple comparisons test), < LOD: read outs below limit of detection.

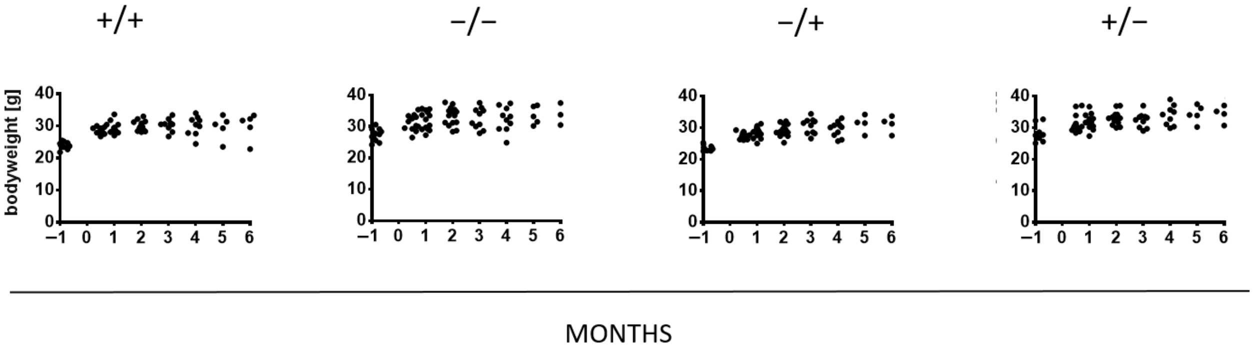

Supplementary Figure S6. Body weight of mice with low-grade persistent inflammatory and intravenously injected PS-IONPs in comparison to control groups. Experimental group: "+/+": animals with low-grade persistent inflammation state (zymosan: 7 cycles á 18 µg/kg body weight, PS-IONPs: 50 µmol Fe/kg body weight). Control groups: "−/−": animals without low-grade persistent inflammation state and no intravenous injection with PS-IONPs, "−/+": animals without low-grade persistent inflammation state but with intravenous injection of PS-IONPs, "+/ −": animals with persistent inflammation but without intravenous injection of PS-IONPs.

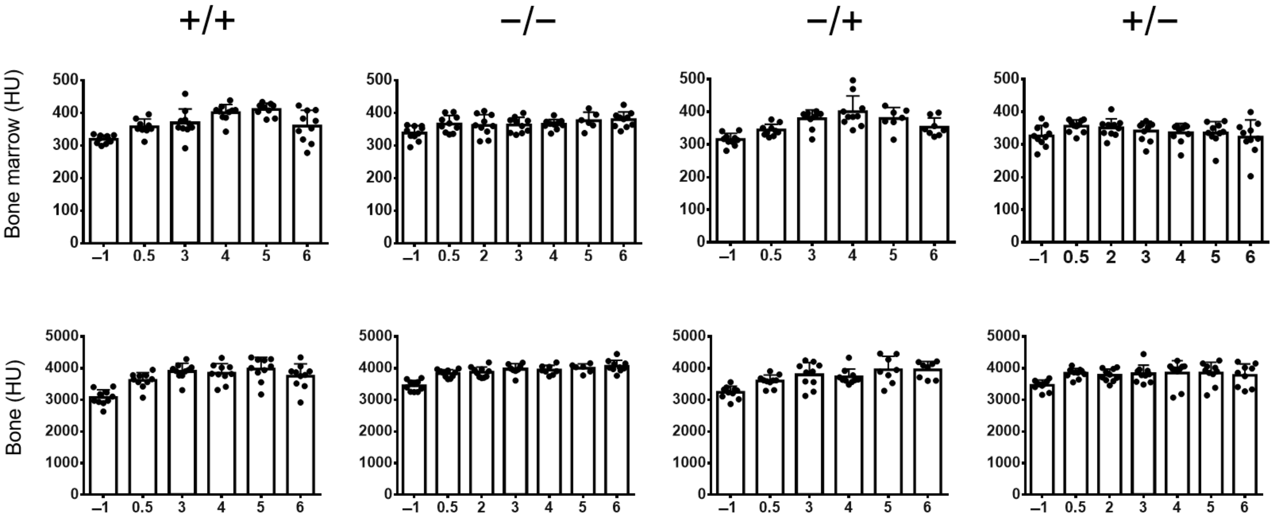

Supplementary Figure S7. X-ray density of bone marrow and bones of animals with persistent inflammatory and intravenously injected PS-IONPs in comparison to control groups. Experimental group: "+/+": animals with low-grade persistent inflammation state (zymosan: 7 cycles á 18 µg/kg body weight, PS-IONPs: 50 µmol Fe/kg body weight). Control groups: "−/−": animals without low-grade persistent inflammation state and no intravenous injection with PS-IONPs, "−/+": animals without low-grade persistent inflammation state but with intravenous injection of PS-IONPs, "+/−": animals with persistent inflammation but without intravenous injection of PS-IONPs. Data are plotted as Hounsfield units (HU). Bones (femur): n = 4 ROIs á 0.2 mm^2 each, 37 pixels; bone marrow: n = 1 ROIs from 0.62 to 5.21 mm^2 depending on the CT-slice intersection plane, between 116 and 977 pixels.

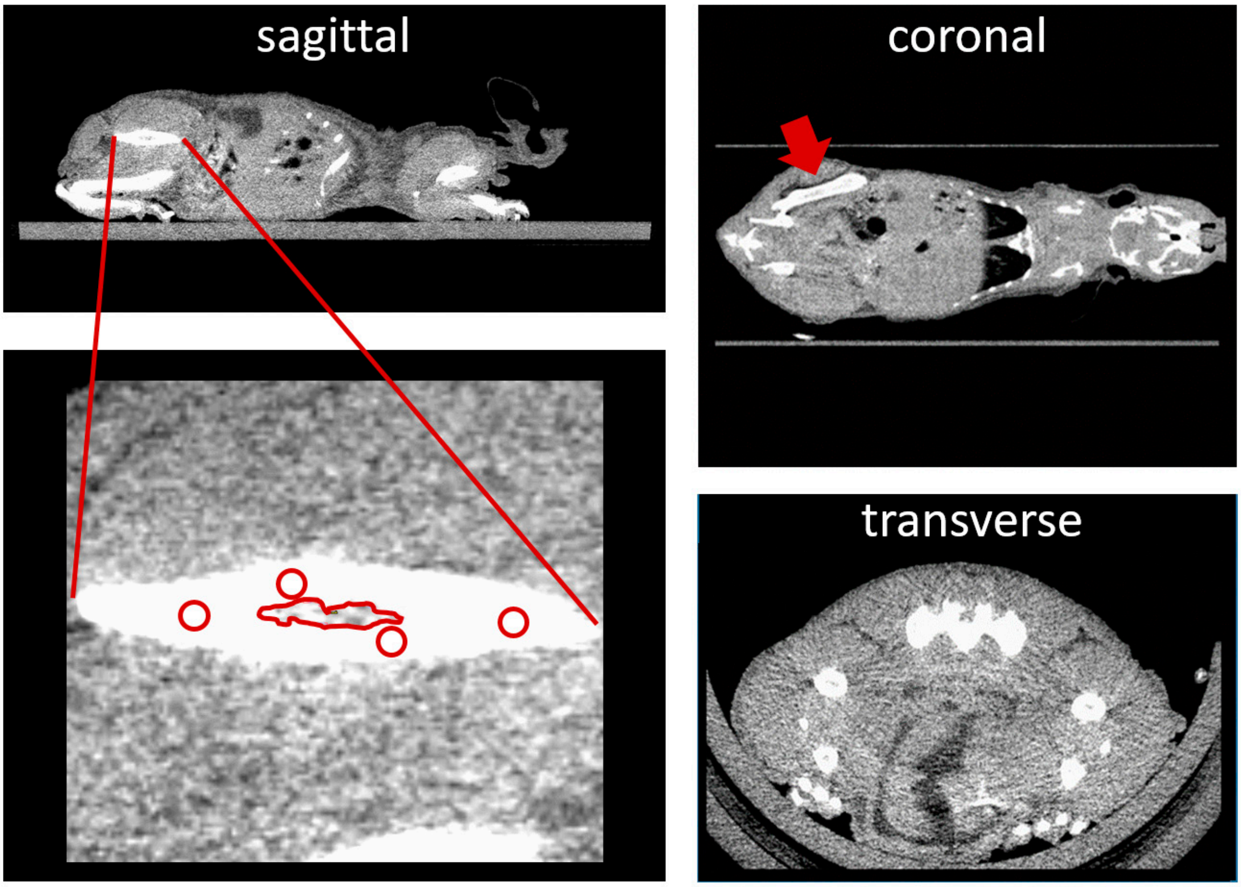

Supplementary Figure S8. Typical CT pictures of different mouse body planes obtained from in vivo mCT imaging (TomoScope® Synergy Twin) using a standard protocol with an x-ray dose of 65 kV. Regions of interest (ROI) were placed on a) bones: n = 4 x spherical ROIs á 0.2 mm^2 each, 37 pixels; b) Bone marrow: one hand-drown ROI from 0.62 to 5.21 mm^2 depending on the CT-slice intersection plane, between 116 and 977 pixels. Red lines: projection of the femur from the sagittal plane into a separate figure, and red arrow: localization of the femur in the coronal plane of the body.

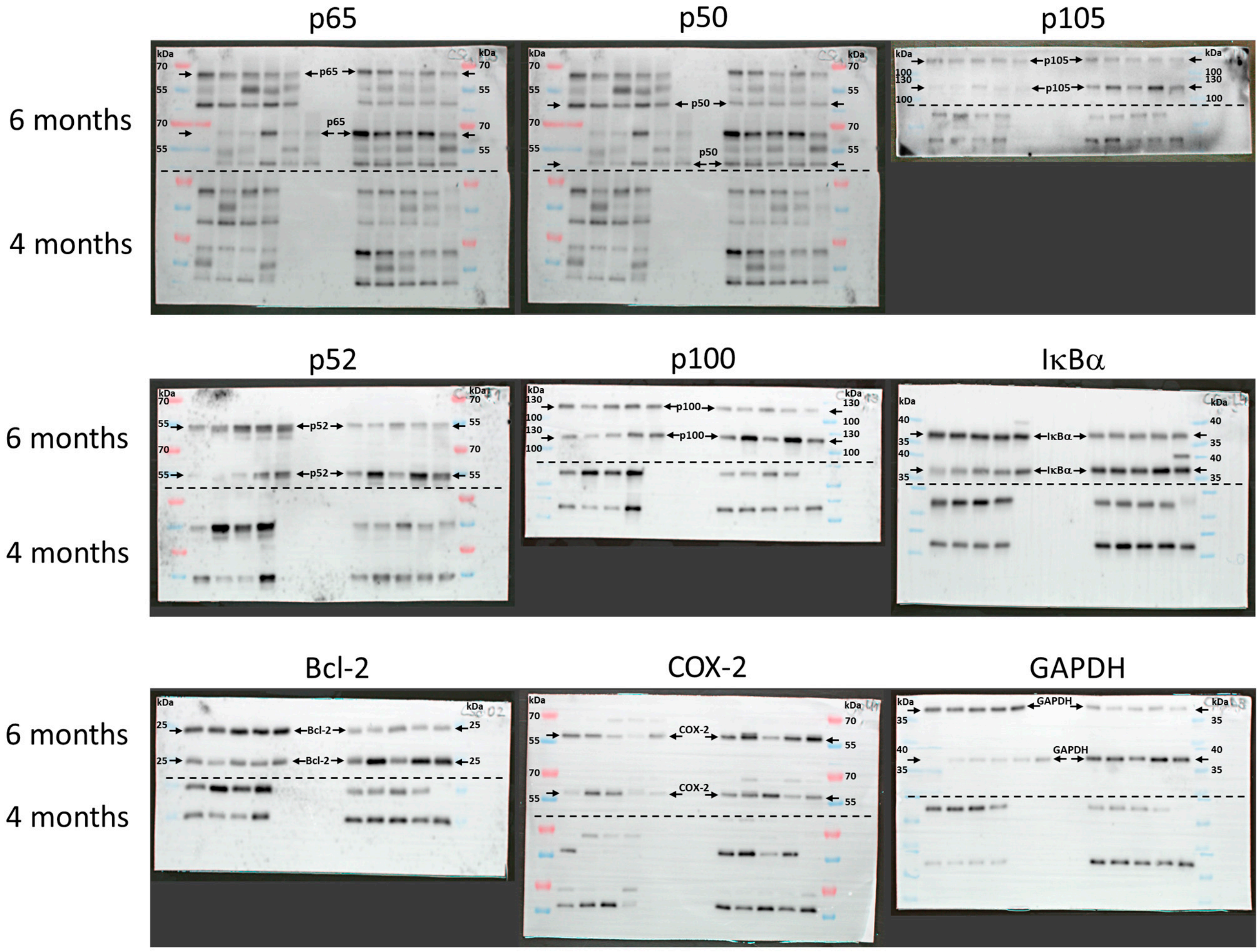

Supplementary Figure S9: Exemplary unadjusted and uncropped pictures of immunoblotting membranes showing the same protein bands from supplementary Figure 3, which were gained from liver lysates from animals after 6 months of intravenous application of PS-IONPs. Because multi-strip Western blotting was used, the membranes also contain protein bands corresponding to the post-observation time of 4 months. Additional data will be available upon request.
